# Supplementary material for: Brain-Derived 11S Regulator (PA28αβ) Promotes Proteasomal Hydrolysis of Elongated Oligoglutamine-Containing Peptides
Source: Int J Mol Sci. 2023 Aug 26;24(17):13275. doi: 10.3390/ijms241713275 (PMC10487437; doi:10.3390/ijms241713275)
Supplement: Supplementary file 1 [file ijms-24-13275-s001.zip › ijms-2533275-supplementary.pdf]

Supplementary Materials

Characterization of purified proteasomes

The core 20S proteasome contains 6 proteolytic sites in total: 2 with caspase-like activity (β1), 2 with trypsin-like activity (β2) and 2 with chymotrypsin-like activity (β5) which allows cleavage after acidic, basic and hydrophobic residues, respectively. By measuring the rates of hydrolysis of 3 different fluorescent substrates (Z-Leu-Leu-Glu-AMC, Ac-Arg-Leu-Arg-AMC, Suc-Leu-Leu-Val-Tyr-AMC), we estimated proteolytic activity of each type in isolated proteasomes. Both 20S and 26S demonstrated significant activity with each of 3 substrates (Table S1). To confirm that proteolytic activity was due to the presence of active proteasome particles, and not due to contamination by other cellular proteases during purification process, we conducted an inhibitory analysis with selective proteasome inhibitor bortezomib and another proteasomal inhibitor MG-132, though latter is also known to inhibit proteases calpain 1 and cathepsin B [48] (Table S1). We found that proteolytic activity was abrogated after addition of proteasome inhibitors to reaction mixture for both 20S and 26S. This result proves that our isolated 20S and 26S proteasomes are free of other proteases. To detect the presence of uncapped 20S particles lacking 19S regulator in purified 26S proteasome, we conducted our activity assay in the presence of 0.02% SDS. Addition of detergent facilitates the opening of the entry channel of 20S proteasome and alleviates the entrance of peptide substrates into proteolytic chambers which leads to increase in proteolytic activity. This effect is not observed with 26S proteasome since binding of 19S to 20S core particle already regulates the opening of this channel in ATP-dependent way [49]. Instead, proteolytic activity of 26S was completely inhibited after addition of 0.02% SDS.

The addition of 11S activator protein to 20S proteasome drastically enhanced cleavage of all three substrates. Importantly, as expected, 11S protein did not activate the hydrolysis of short peptide substrates by 26S proteasome.

Table S1. Peptidase activity of the samples of 20S and 26S proteasome from murine brain.

Proteasome, additive	SPECIFIC PEPTIDASE ACTIVITY, nM/MIN/μg USING STANDARD SUBSTRATE		
	**Suc-LLVY-AMC**	**Ac-RLR-AMC**	**Z-LLE-AMC**
20S	2.37 ± 0.13	2.78 ± 0.07	1.14 ± 0.02
20S+0.5 μM Bortezomib	0.0255 ± 0.0007	-	-
20S+0.1 μM MG132	0.093 ± 0.003	-	-
20S+0.02% SDS	34 ± 6	-	-
20S+11S (1:2, mole:mole)	8.8 ± 0.4	8.4 ± 0.2	5.2 ± 0.1
26S	23.8 ± 0.8	1.92 ± 0.06	5.4 ± 0.1
26S+0.5 μM Bortezomib	0.48 ± 0.01	-	-
26S+0.1 μM MG132<br>(1 μM MG132)	1.63 ± 0.02<br>(0.33 ± 0.01)	-	-
26S+0.02% SDS	n.d.	-	-
26S+11S (1:2, mole:mole)	26.4 ± 2.5	2.5 ± 0.2	6.7 ± 0.5

Values are represented as means ± SEM of three independent experiments run in duplicate. The molar ratio between the proteasome and 11S regulator protein was 1:2.

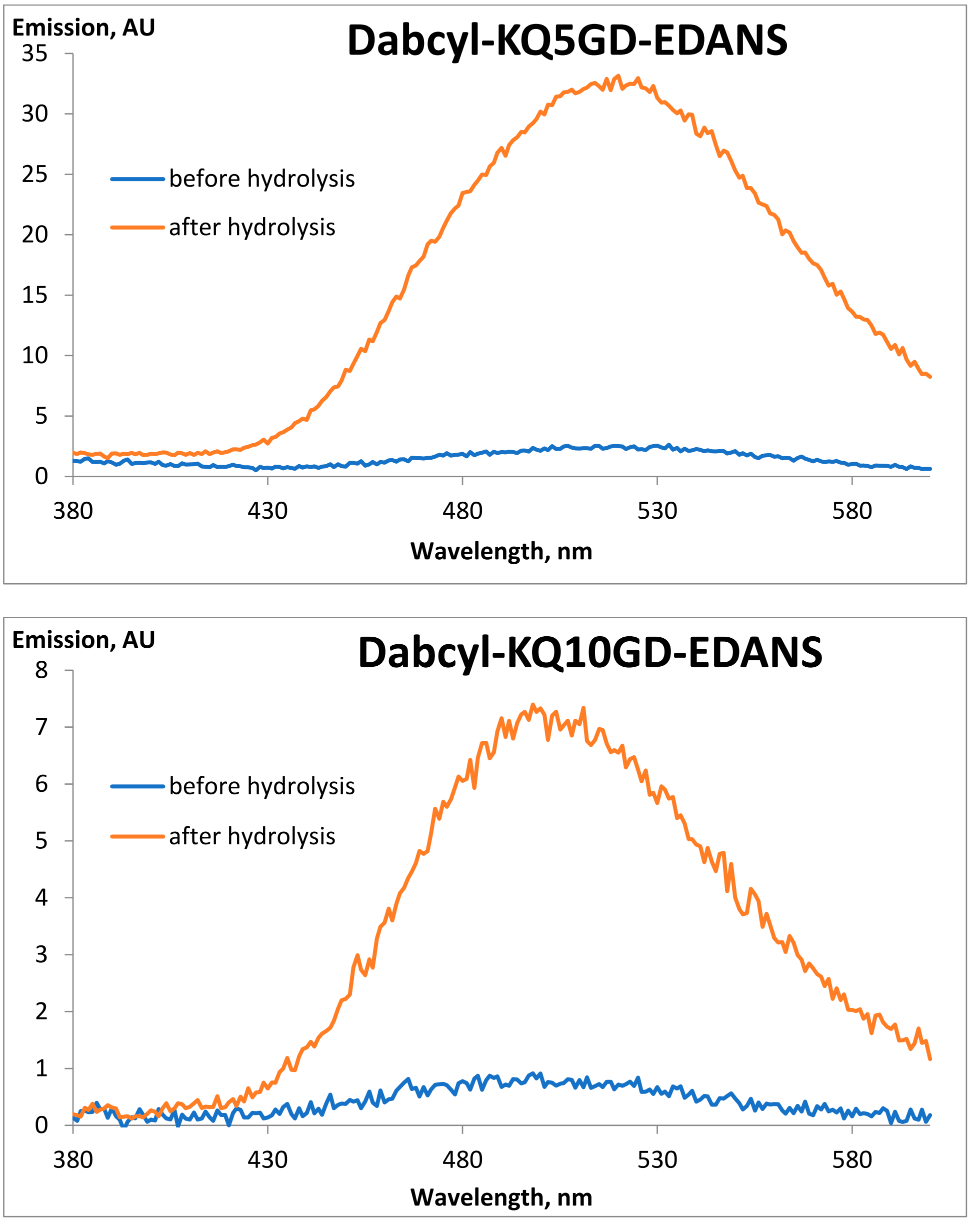

Figure S1. Fluorescence spectra of peptide substrates before and after complete hydrolysis by subtilisin in 50 mM Tris-HCl buffer, pH 8. Emission wavelength was fixed at 340 nm.

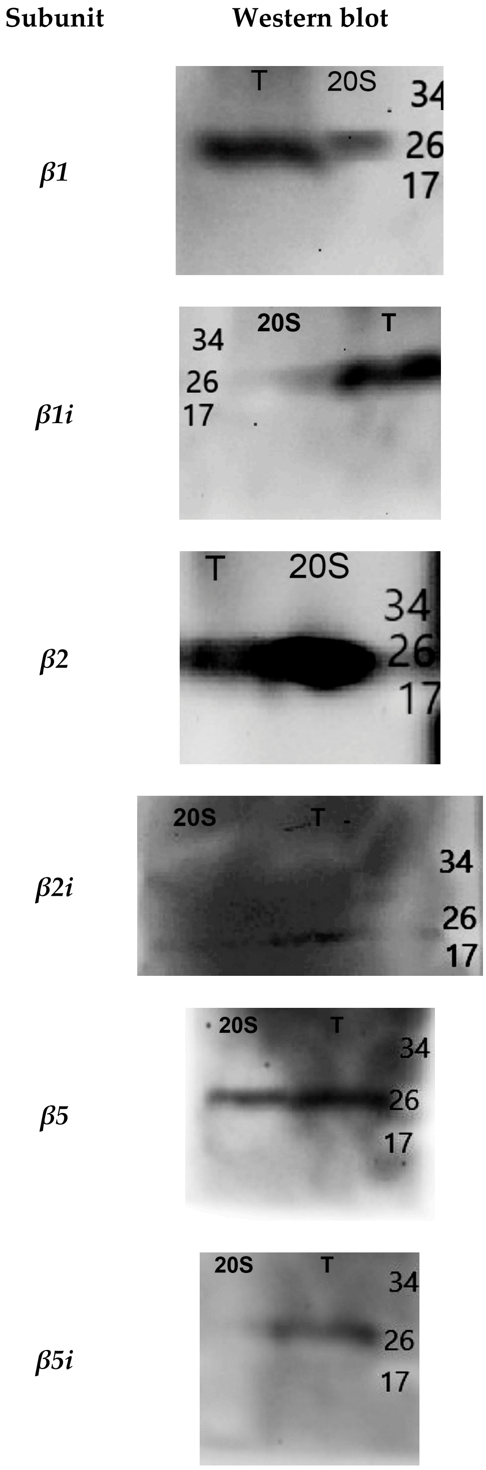

Figure S2. Catalytic subunit composition of isolated and purified 20S proteasome isolated from mice brain analyzed by western blot. Mouse thymus homogenate was used as positive control, marked by T.

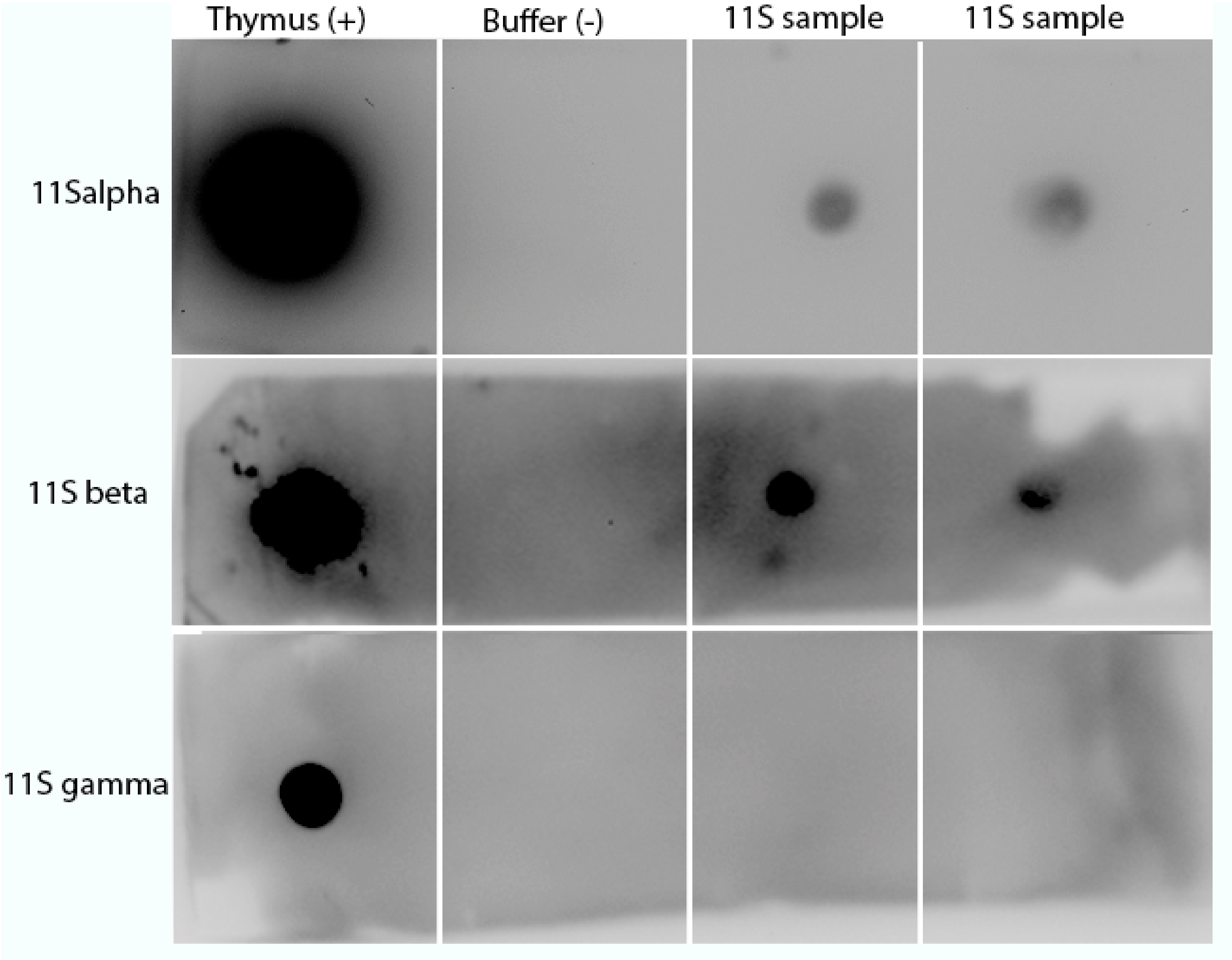

Figure S3. Dot-blot of isolated and purified 11S proteasome regulator. Mouse thymus homogenate was used as positive control, Tris-HCl buffer was used as negative control. The samples were dotted onto the membrane, dried, then the membrane was blocked and treated with antibodies to 11Sα (upper panel), or 11Sβ (middle panel), or 11Sγ (bottom panel).

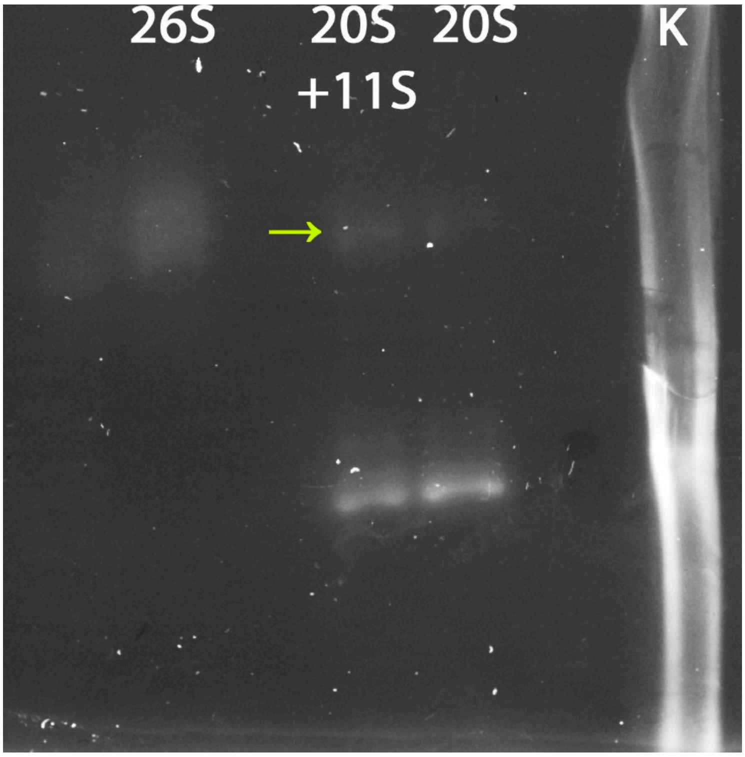

Figure S4. Native electrophoresis in PAGE of 20S and 26S proteasome with or without addition of 11S regulator. The purified preparations of the 20S proteasome was mixed with the 11S-regulatory protein in a molar ratio of 1:2, respectively. The activity of the 20S- and 26S-proteasome was detected at λ = 366 nm after gel treatment with a solution of Suc-LLVY-AMC (150 μM in buffer E). A conjugate of dansyl chloride with thyroglobulin (~700 kDa) was used as a molecular weight marker (K, right lane). The arrow marks the activity zone corresponding to the 20S+11S complex.

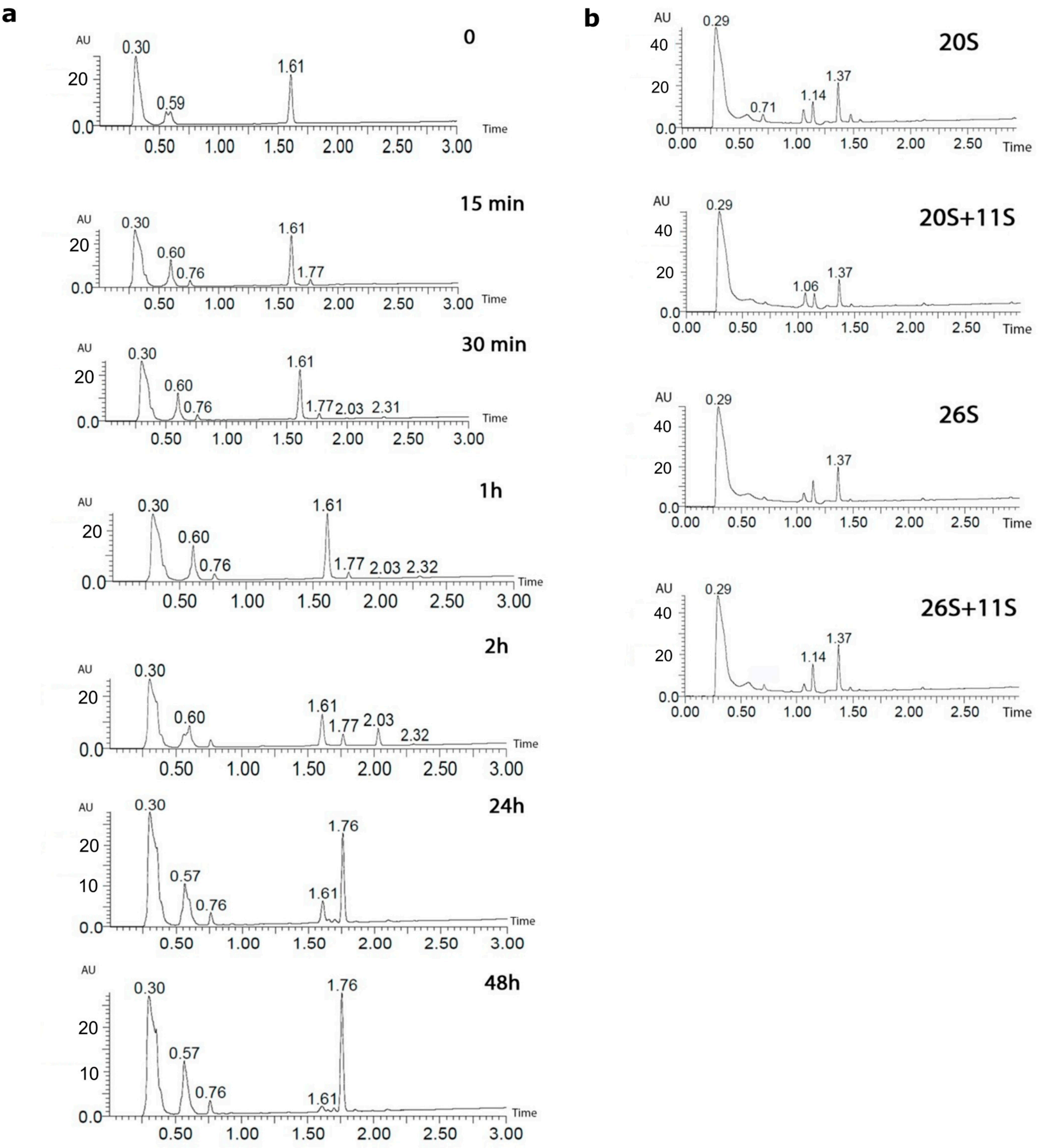

Figure S5. LC/MS analysis of (**a**) time-course of Dabcyl-KQ5GD-EDANS hydrolysis by the 20S proteasome (**b**) samples of Dabcyl-KQ10PPD-EDANS hydrolysis by 20S and 26S proteasome with or without addition of 11S regulator after 24h of incubation.

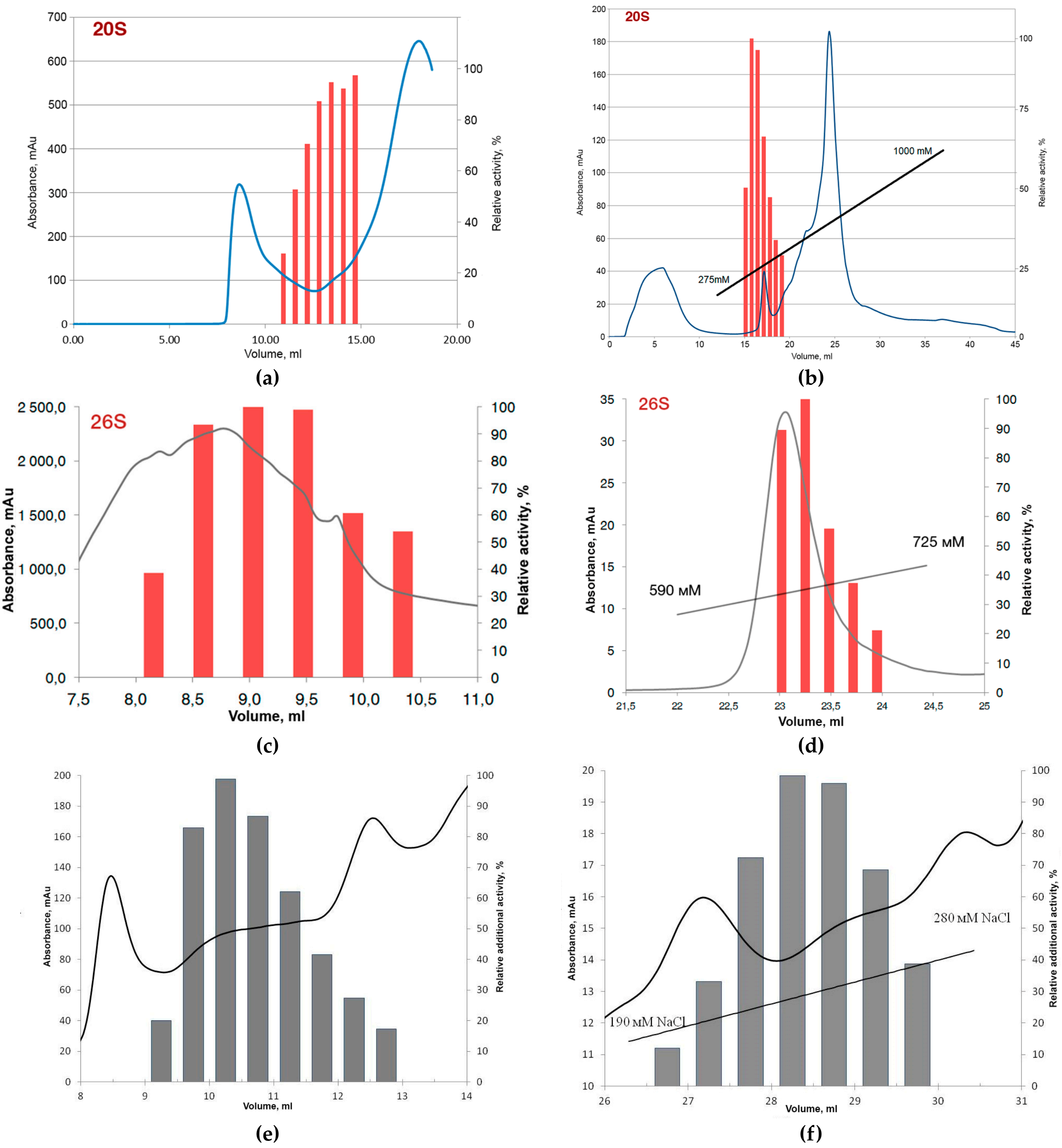

Figure S6. Chromatographic profiles for 20S proteasome, 26S proteasome and 11S regulator purification by gel filtration (**a**,**c**,**d**) and ion_exchange chromatography (**b**,**d**,**f**). The relative activities of the selected fractions towards the Suc–Leu–Leu–Val–Tyr–AMC substrate are presented as columns. The NaCl concentration gradient in the eluent is shown by a slanting line. Superose6™ was used for gel filtration of 20S and 26S proteasomes and Superdex200™ was used for 11S regulator. MonoQ™ was used for Ion-exchange chromatography of 20S and 26S proteasome, DEAE-Sepharose was used for 11S regulator. For more experimental detail see section 4.2. *Isolation of proteasome particles.*
